# A Comprehensive Review of Fc Gamma Receptors and Their Role in Systemic Lupus Erythematosus

**DOI:** 10.3390/ijms26051851

**Published:** 2025-02-21

**Authors:** Jesús Sepúlveda-Delgado, Luis Llorente, Susana Hernández-Doño

**Affiliations:** 1 Research Division, Servicios de Salud IMSS BIENESTAR, Hospital Regional de Alta Especialidad Ciudad Salud, Tapachula 30700, Mexico; jesussd52@gmail.com; 2 Department of Immunology and Rheumatology, Instituto Nacional de Ciencias Médicas y Nutrición Salvador Zubirán, Mexico City 14000, Mexico; 3 Physiology and Pharmacology Department, Chemistry and Pharmacy Faculty, Universidad de El Salvador, San Salvador 01101, El Salvador

**Keywords:** Fc gamma receptor, FcγR, FcgR, FcgRIIa, FcgRIIIa, FcgRIIIb, SLE, phagocytosis, autoimmune disease, autoimmunity

## Abstract

Receptors for the immunoglobulin G constant fraction (FcγRs) are widely expressed in cells of the immune system. Complement-independent phagocytosis prompted FcγR research to show that the engagement of IgG immune complexes with FcγRs triggers a variety of cell host immune responses, such as phagocytosis, antibody-dependent cell cytotoxicity, and NETosis, among others. However, variants of these receptors have been implicated in the development of and susceptibility to autoimmune diseases such as systemic lupus erythematosus. Currently, the knowledge of FcγR variants is a required field of antibody therapeutics, which includes the engineering of recombinant soluble human Fc gamma receptors, enhancing the inhibitory and blocking the activating FcγRs function, vaccines, and organ transplantation. Importantly, recent interest in FcγRs is the antibody-dependent enhancement (ADE), a mechanism by which the pathogenesis of certain viral infections is enhanced. ADEs may be responsible for the severity of the SARS-CoV-2 infection. Therefore, FcγRs have become a current research topic. Therefore, this review briefly describes some of the historical knowledge about the FcγR type I family in humans, including the structure, affinity, and mechanism of ligand binding, FcγRs in diseases such as systemic lupus erythematosus (SLE), and the potential therapeutic approaches related to these receptors in SLE.

## 1. Introduction

**Figure 1:**
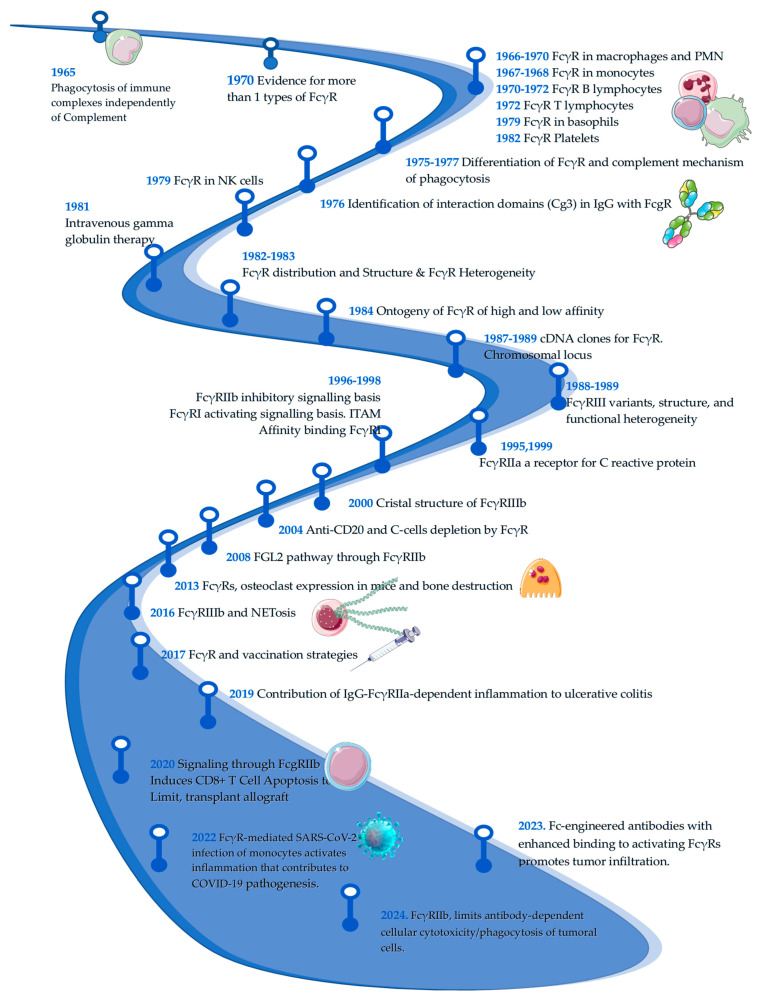
Timeline of Fcγ receptors research. The figure below shows a timeline on the research of Fc gamma receptors. It is a basic and informative line showing the evolution of research on the subject; important points may not have been included in the figure. Dates and references: 1965 [7], 1966 [8], 1967 [9], 1968 [10,11], 1970 [12,13,14], 1972 [15], 1975 [16], 1976 [17], 1977 [18], 1979 [19], 1980 [20], 1982 [21], 1983 [22], 1984 [6], 1988 [23,24], 1989 [25,26,27], 1995 [28], 1996 [29], 1998 [30], 1999 [31], 2000 [32], 2004 [33], 2008 [34], 2013 [35], 2016 [36], 2017 [37], 2019 [38], 2020 [39], 2022 [40], 2023 [41], 2024 [42].

Human Fc receptors for IgG (FcγR) constitute a family of receptors that are genomically located on the long arm of chromosome 1 in band 1.21 and 1.22 [1,2,3,4,5]. FcγRs are widely distributed in almost all immune cells. These receptors exert diverse functions through engagement with the Fc fraction of immunoglobulin G complexes, which are canonical ligands [6]. The ability of FcγRs to engage IgG Fc fragments allows responsiveness to all antigens opsonized with IgG. This versatility gives FcγRs a pivotal function in host defense and clearance of immune complexes. However, an alteration in FcγR function could result in impaired host defense or autoimmunity. As a result, FcγRs have become a key group of receptors, the variants of which are related to susceptibility or protection against autoimmune diseases. In addition, FcγRs are currently considered pharmacological targets of foremost importance. The engineering of Fc fragments of monoclonal antibodies(mAbs) aims to improve the performance of and enhance binding of mAbs to FcγRs. The study of FcγRs is a necessary and promising field of research. Hence, this review aims to bring together the essentials of the research timeline and immunobiology of these receptors that are known to date (Figure 1), their role in autoimmune diseases, with emphasis on systemic lupus erythematosus, and their role as mediators of pharmacological responses. The idea for this review arose from the desire to gather the elementary information that a scientist needs to know if he or she is just starting out in the study of FcγR and SLE.

## 2. FcγRs Classification, Function, Variants and Role in SLE Pathology

**Figure 2:**
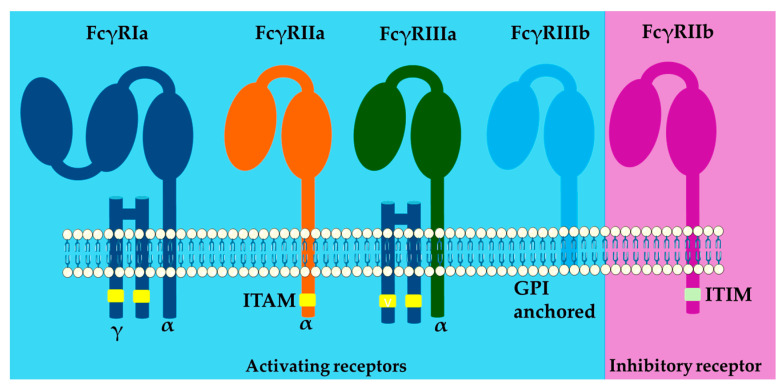
Structure of Fc gamma receptors. Fc receptors are composed of an alpha backbone (α), where the activation domain is located; the immunoreceptor tyrosine-based activation motifs (ITAMs), as is the case for FcγRIIa. For FcγRIa and FcγRIIIa, there are accessory chains, such as gamma (γ) and others, which are the carriers of the ITAMs necessary for signaling. The only inhibitory receptor, FcγRIIb, contains immunoreceptor tyrosine-based inhibitory motifs (ITIMs). Each receptor is composed of two immunoglobulin-like domains, with the exception of FcγRIa, which is composed of three domains that favor high-affinity characteristics.

Human FcγRs are members of the immunoglobulin gene superfamily and can be distinguished based on size, affinity for ligands, primary structure, ligand specificity, and monoclonal antibody reactivity [27,43]. However, canonical type I FcγRs are generally classified as activating or inhibitory depending on the signaling properties of their intracellular domains (Figure 2). The most important activating FcγRs include FcγRI (CD64), FcγRIIa (CD32a), and FcγRIIIa (CD16a), which contain or associate with immunoreceptor tyrosine-activating motifs (ITAM) [44,45]. In contrast, FcγRIIb(CD32b) is the sole inhibitory FcγR that mediates signaling through an immunoreceptor tyrosine inhibitory motif (ITIM) [46]. In contrast to activating or inhibitory FcγRs, FcγRIIIb(CD16b) is expressed as a glycosyl phosphatidyl inositol-anchored (GPI) protein and is therefore incapable of signal transduction alone because it associates with activating receptors (such as FcγRIIa) to display a functional outcome [47]. Affinity is another broad classification criterion; FcγRI is the sole FcγR that engages monomeric IgG with high binding affinity [48]. Other FcγRs exhibit low affinity for monomeric IgGs but high affinity for multimeric IgG immune complexes (ICs) or opsonized cells [49].

Molecular cloning and sequence analysis of cDNAs encoding human FcγRI, FcγRII, and FcγRIII have indicated that they are structurally related and contain conserved extracellular ligand-binding regions of Ig-like domains and, as such, belong to the Ig superfamily [4,23,50,51,52,53,54,55].

### 2.1. FcγRI(CD64)

*Structure:* FcγRI is a type 1 transmembrane glycoprotein of ~70-kDa. FcγRI is structurally distinct and contains an extracellular immunoglobulin interactive region of three extracellular Ig-like domains in contrast to the two domains of the low-affinity receptors FcγRII and FcγRIII (Figure 2) [50,56]. The third extracellular domain is different, whereas the first two domains are homologous to the extracellular domains of FcγRII and FcγRIII. The unique IgG-binding characteristics of FcγRI are conferred by domain three. Although this domain is not essential for Fc binding, it determines the specific high-affinity interaction between FcγRI and IgG2a [57]. The interaction between domains 2 and 3 of FcγRI and domain 1 plays a supporting role in maintaining the conformational stability of the receptor [30,58]. Moreover, FcγRI highlights a unique glycan recognition mechanism that adds structurally improved affinity [48].

*Functions:* FcγRI is predominantly expressed in myeloid cells, including monocytes, macrophages, neutrophils, and dendritic cells. Previous studies have indicated that FcγRIa plays a significant role in neutrophil recruitment during acute infectious diseases [59]. However, FcγRI is a unique FcγR that engages monomeric IgG with high binding affinity, which means that this receptor does not require immune complexes to activate the signaling pathway [48].

*Role in SLE:* Some studies have shown that monocyte surface expression of FcγRI correlates with type-I interferon levels in SLE [60]. The expression of FcγRI is increased in SLE and even more so in lupus nephritis. Additionally, FcγRI expression is positively associated with serum creatinine levels and indicators of systemic inflammation. 

Monocytes from patients with high FcγRI expression also exhibited increased chemotaxis and capacity to produce monocyte chemotactic protein 1 (MCP-1) [61]. Recent studies have demonstrated that FcγRI is an essential component in the response of human neutrophils to immune complexes leading to the production of ROS, MCP-1, and degranulation, which may help explain how neutrophils contribute to tissue damage associated with immune complex-associated disease, such as lupus [62].

### 2.2. FcγRII(CD32)

Structure: FcγRII isoforms FcγRIIa and FcγRIIb are type 1 transmembrane glycoproteins of ~40 kDa that contain extracellular regions of two Ig-like domains. The extracellular and transmembrane domains are highly conserved, and both isoforms display nearly identical ligand-binding domains, yet their intracytoplasmic regions differ: FcγRIIa contains ITAM and FcγRIIb contains ITIM, giving an antagonist functional outcome (Figure 2) [63].

#### 2.2.1. FcγRIIa

FcγRIIa is probably unique to higher primates, the most widespread in immune cells, and is the major phagocytic FcγR in humans [64]. 

*Functions:* FcγRIIa is a prototype phagocytic receptor belonging to the FcγR family. However, its function depends on the cells in which the receptor is expressed; macrophages and neutrophils show high efficiency of phagocytic activity through this receptor [65].

*Single nucleotide variants:* Because FcγRIIa is widely distributed in immune cells, single nucleotide variants (SNVs) that affect affinity ligand binding have been extensively studied. The most widely studied example is the change in arginine (R) by histidine (H) at position 131. Individuals homozygous for the R allelic form of FcγRIIa are more susceptible to bacterial infections and autoimmune diseases than those homozygous and heterozygous for the H allelic form of FcγRIIa [66,67]. Binding studies using Ig fusion proteins of FcγRIIa alleles showed that the R allele has significantly lower binding affinity to IgG2, IgG1, and IgG3 subtypes [68]. The three-dimensional structure of the complex between both variants and the Fc region of humanized IgG1 has shown affinity binding differences mainly at the hinge level [64].

*Role in SLE:* It has been demonstrated that the mechanism of neutrophil activation in the pathogenesis of SLE requires DNA and RNA immune complexes (ICs), and this requires FcγRIIa engagement. SLE-derived ICs activate neutrophils to release ROS and chemokines in an FcγRIIa-dependent manner. This has been demonstrated through assays blocking FcγRIIa, which inhibits ROS release from these cells. Dysregulation or activation of FcγRIIa in patients with SLE can contribute to the overproduction of autoantibodies, immune complex formation with consequent organ damage, and excessive inflammation that induces flares [69].

#### 2.2.2. FcγRIIb

FcγRIIb is the sole inhibitory FcγR that confers to this receptor a different role in the modulatory scheme of Fcγ-activating receptors [70].

*Functions:* On innate immune cells, the inhibitory function of FcγRIIb directly antagonizes the activation of FcγRs; therefore, it equilibrates the cellular outcome, generating an inhibitory balance and attenuating the activation signaling, such as co-signaling molecules [39]. More importantly, this receptor crosslinks with the B-cell receptor (BCR), shaping the B repertoire of lymphocytes and inducing apoptosis in autoreactive plasma cells. Moreover, FcγRIIb signaling controls antibody levels involving the differential expression of the receptor on B cell subpopulations in which FcγRIIb functions independently of the BCR to eliminate antibody-secreting effector cells and inhibit naïve B cell proliferation without compromising long-lived antigen-specific memory B cells [71,72].

*Single nucleotide variants:* Several polymorphisms have been described in FcγRIIb. The most important variants affect inhibitory capability. The most studied variants in the transmembrane domain are FcγRIIb isoleucine (I) with threonine (T) at position 187 and isoleucine with threonine at position 232. The FcγRIIb 187T variant is known to be excluded from lipid rafts and has decreased inhibitory potential toward BCR signaling [73,74,75]. Likewise, the FcγRIIb 232T variant decreases affinity to lipid rafts (this prevents interaction of FcγRIIb with ITAM-containing receptors, such as the activating FcγR and the BCR) and attenuates inhibitory effects on B cell receptor signaling [76]. The haplotype -386C/-120A (known as 2B.4, which is the less frequent haplotype) in the promoter confers an increased transcription of the receptor [77,78]. The haplotype 2B.4 allows the novo FcγRIIb expression on neutrophils and monocytes [79], which allows a modulatory effect.

*Role in SLE:* FcγRIIb T232I (rs1050501) leads to decreased suppressor activity, thereby enhancing the susceptibility to SLE. These genotype and allele frequencies of FcγRIIb are associated with incidence of leukopenia, rash, mucosal ulcer, arthritis, and thrombocytopenia in SLE patients, these parameters are considered in the SLE Disease Activity Index (SLEDAI), the main clinimetric tool to evaluate the remission and low disease states [80]. 

Therefore, FcγRIIb 232T is a dysfunctional receptor. Monocyte-derived macrophages from SLE patients with the 232T genotype showed increased FcγR-mediated vascular endothelial growth factor A (VEGF-A) production. Thus, ICs contribute to inflammation through VEGF-A-driven lymph node lymphangiogenesis, which is controlled by FcγRIIb [81].

Furthermore, the haplotype 2B.4, in the promoter has been associated with susceptibility to SLE in Europe, and paradoxically, confers protection against the development of lupus nephritis [77,78]. 

The importance of this receptor in SLE is such that lupus-like mice models are generated with FcγRIIb knock-out [82]. In these animal models, it has also been shown that inflammatory systemic conditions, such as obesity, allergy, or conditions that can induce leaky gut, such as NSAIDs and alcohol, can cause permeability and endotoxemia, which can induce or worsen autoimmunity in the absence of the modulation/inhibition exerted by FcγRIIb. Specifically, obesity facilitates lupus onset and exacerbates lupus activity, partly through saturated fatty acid-induced gut barrier defects and systemic inflammation. Allergy makes dendritic cells more susceptible to hyperactivation, which activates lupus nephritis, as indicated by anti-dsDNA, proteinuria, and renal immune complex deposition. In NSAID enteropathy, mitochondrial function and cytokine production in macrophages are more prominent. Hence, lupus disease activation due to NSAID enteropathy-induced gut leakage is possible. Finally, alcohol induces more prominent liver damage and actives lupus-like characteristics [83,84,85].

### 2.3. FcγRIII 

*Structure:* There are two functional isoforms of FcγRIII. Human FcyRIII is heterogeneous in size, with a molecular weight ranging from 50 to 80 kDa [21,24,86]. This heterogeneity is due to the extensive N-linked glycosylation of two distinct isoforms, FcyRIIIa and FcyRIIIb [50]. A single amino acid change determines the intracellular domain differences between FcyRIIIa and FcγRIIIb isoforms. Human FcyRIIIb contains Se203, which specifies a glycosyl-phosphatidylinositol (GPI) linked molecule, whereas FcyRIIIa contains Phe203, which disrupts the signal for the formation of a GPI anchor, thus preserving the transmembrane and cytoplasmic tail and producing a transmembrane molecule (Figure 2). Both are activating receptors and have different association requirements to display effective signaling [50].

#### 2.3.1. FcγRIIIa

FcγRIIIa is an activating receptor that is recognized by antibody-dependent cellular cytotoxicity (ADCC) function. The FcγRIIIa protein is expressed as a transmembrane protein on monocytes, tissue specific macrophages, dendritic cells, δ/γT cells, and natural killer cells [70].

*Functions:* ADCC is an Fc-dependent effector function of IgG that is important for antiviral immunity and antitumor therapies. NK cells mediate ADCC through the binding of antibody-opsonized target cells by membrane-expressed FcγRIIIa, and induce cytotoxicity by releasing granzymes and perforins stored in intracellular granules. This mechanism contributes to the killing of tumor cells during immunotherapy. NK cell-mediated ADCC is mainly triggered by IgG-subclasses IgG1 and IgG3 through the IgG-Fc-receptor FcγRIIIa [87].

*Single nucleotide variants:* The most important variants are related to the development of autoimmune diseases. The best known is FcγRIIIa (rs396991) valine (V) by phenylalanine(F) at position 158. This change affects the receptor affinity. The increased binding capacity of the 158V allele results in more robust downstream functional effects [88].

*Role in SLE:* FcγRIIIa 158F, the allele with lower affinity, is associated with SLE susceptibility in different ethnic groups [89]. However, FcγRIIIa 158V is associated with severity and progression to the final stages of renal involvement in SLE. This is consistent with the fact that FcγRIIIa 158V displays higher binding affinity to IgG1, IgG3, and IgG4 consistent with the functional outcome of this receptor, promoting vigorous local inflammatory responses [90]. Case–control analyses have generated evidence that differs in the association of this polymorphism and SLE, so ethnicity and the triggers of the environment are important to be considered in the background so as not to generalize the role of the variants of this receptor. FcγRIIIa could induce over inflammation through the interaction with immune complexes, with the consequent excessive activation of immune cells. The altered function of FcγRIIIa could affect immune cells’ ability to eliminate immune complexes, contributing to their accumulation, enhancing organ damage, and increasing the flares and recurrence of symptoms. 

#### 2.3.2. FcγRIIIb

FcγRIIIb is a unique receptor in the FcγR family that is anchored to the outer leaflet of the plasma membrane by a GPI moiety whose surface expression is 10-fold higher than that of FcγRIIa (135,000 versus 10,000 receptors/cell, respectively) [91]. Because of this difference in anchoring FcγRIIIb to the membrane, it does not have intracellular signaling motifs.

*Functions:* FcγRIIIb cooperates with other FcγRs to promote phagocytosis of antibody-opsonized microbes by favoring Ca^2+^ influx [91]. Additionally, FcγRIIIb induces a neutrophil extracellular trap-producing phenotype in the absence of activation of FcγRIIa [36]. 

**Figure 3:**
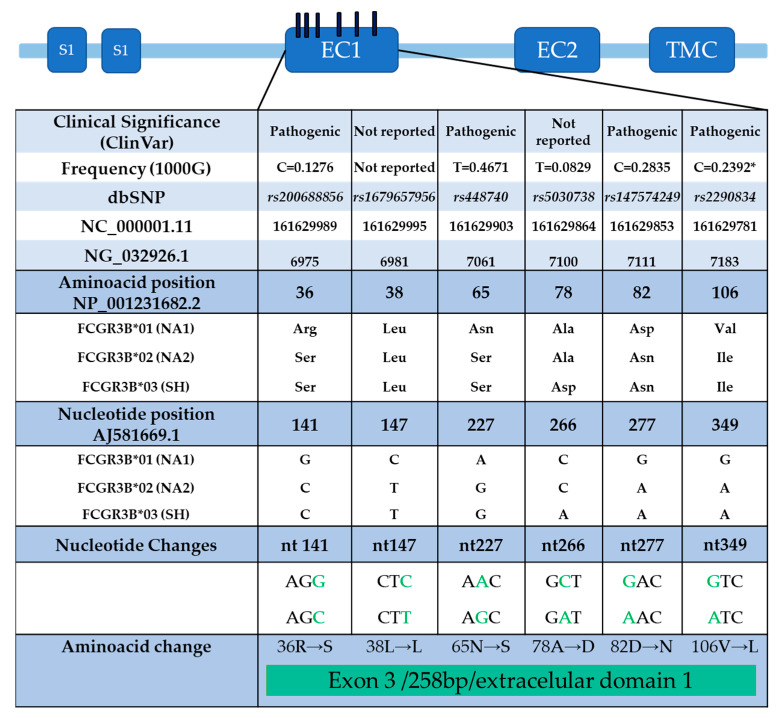
FcγRIIIb receptor polymorphism distribution in exon 3 (EC1): The positions of the nucleotides that have changes can be distinguished according to AJ581669.1, which was generated and used in the initial studies of the receptor. The classification of clinical significance (ClinVar) is also shown, and some cases considered pathogenic to date are under review. The frequency of the variant, according to the database of the 1000 genomes; the changes, according to the single nucleotide variants database; and the name of the variant, according to the global database of all SNPs, are shown. Additionally, the NCBI Reference Sequence Database (RefSeq) is included to identify the location of variants on the chromosome (NC_000001.11), gene (NG_032926.1), protein (NP_001231682.2), and mRNA (AJ581669.1). Modified from [90].

*Single nucleotide variants:* There are three known alleles: FCGR3B*01 (NA1, which means neutrophil antigen 1), FCGR3B*02 (NA2), and FCGR3B*03 (SH). Alleles FCGR3B*01 and FCGR3B*02 differ by five nucleotides at positions 141, 147, 227, 277, and 349 of exon 3, and FCGR3B*03 differs from FCGR3B*02 by only one nucleotide at position 266 of exon 3. The allele polymorphism of FcγRIIIb appears to modify neutrophil phagocytosis (Figure 3) [90,92].

*Role in SLE:* The literature displays conflicting results regarding the association between SLE susceptibility and FcγRIIIb polymorphisms, even in studies with the same genetic background. This may be because some typing techniques, such as PCR, may not discriminate between the *01 and *02 alleles. However, sequencing studies have associated SLE with the FCGR3B*01 allele, as well as with the FCGR3B*01/*01 and FCGR3B*01/*02 genotypes [92]. On the contrary, another study associates FCGR3B*02 homozygotes with the development of SLE. Additionally, a specific lupus phenotype, lupus nephritis, is more likely to appear in individuals with the genotype FCGR3B*02/*02 [93].

## 3. Ligand Binding 

The suspicion that there were different types of Fcγ receptors and different affinities in ligand binding was considered in 1970, when differences in the response performed by polymorphonuclear lymphocytes and monocytes to the same immune IgG complex were supported, which was later confirmed in 1982 [6,94,95]. Currently, the affinity of FcγRs varies according to the type of IgG, and studies of variants of these receptors have also reported changes in binding affinity (Table 1)**.**

It is well known that the FcγR binds to the Fc fraction of the IgG. However, it has recently been determined that certain pentraxins, such as C-reactive protein and serum amyloid P (SAP) can activate FcγRI and FcγRIIa, favoring phagocytosis activation pathways. Additionally, the most recently described ligand, cytokine fibrinogen-like 2 (Fgl2), can bind to FcγRIIb and induce the caspase-3/7-mediated apoptosis subset of CD8+ T cells [28,31,39,107].

FcγR-IC binding. The elements that regulate the binding of FcγR and IgG immune complexes are the second domain of the FcγR and IgG subtypes [108]. However, differences in the FcγR family have been demonstrated. The high degree of amino acid conservation in the extracellular domains of FcγR and the constant sequence of the IgG Fc fraction has allowed modeling of the mechanism of FcγR-ligand (an immune complex of IgG) binding [109]. One of the best examples described is the binding of human immunoglobulin G to the soluble FcγRIII receptor. It was found was that the contact interface includes several amino acids in the second domain of FcγR, which interact with the Fc fraction of IgG (Cγ2). In the FcγRIII–IgG complex, the extracellular portion of FcγRIII binds asymmetrically to a single IgG molecule. This confirms the 1:1 stoichiometric binding model, which explains why IgG molecules cannot spontaneously trigger FcγR-mediated cellular responses in the absence of cross-linking by multivalent antigens [110]. This model avoids permanent stimulation of the immune system by monomeric immunoglobulins present at high concentrations in serum [32].

However, despite the low variability in the contact site of FcγR with IgG immune complexes, there are consistent differences in binding affinity. The second recognition site is the hinge peptide. Its importance has been demonstrated by introducing mutations that abrogate binding of recombinant soluble FcγRIIa to human IgG1 inmune complexes. Thus, hinge peptide has been linked to variations in the affinities with which FcγR bind to IgG immune complexes [111]. Additionally, FcγRII and FcγRIII are 50% identical, and these differences affect the loops in contact with the hinge, but not the contact regions of Cγ2-A and Cγ2-B. Other examples include SNVs at the hinge peptide level. For instance, the FcγRIIa arginine-131 variant affects the binding affinity. A possible explanation may be steric clashes between the larger side chain of arginine-131 in the receptor and proline-238 of the hinge peptide with associated structural rearrangements [32].

In contrast, FcγRI has a high affinity, allowing it to bind monomeric IgG1. It has been assumed that the significantly higher affinity of FcγRI is mediated by its third domain (which differentiates it from all other receptors of the Fcγ family) because the two N-terminal domains show an affinity for IgG comparable to that of FcγRII and FcγRIII. However, once again, the hinge comes into play; an assay showing the variation in the hinge where glutamic acid (E235) replaces leucine (L235) increases the affinity of mIgG2b by more than 100-fold, underscoring the importance of this residue for FcγRI binding. Therefore, it should be emphasized that the role of the additional FcγRI domain in the enhanced binding of IgG has not been fully elucidated. It can contribute to affinity by stabilizing the open receptor conformation or binding directly to the Fc fragment [32].

Regarding the importance of FcγR affinity, early research has shown that the same ligand triggers different responses in different cell types. SNVs in the binding site have been found to be associated with a variety of autoimmune diseases. Additionally, receptor binding is critical for antibody-based immunotherapy [108]. The binding quality of the IgG Fc fraction to FcγR is important because the interactions of therapeutic antibodies may be affected by various normal stresses, a consequence of their administration in vivo. This type of analysis aims to be turned into a quality test to deliver an antibody with an effective affinity in in vivo scenarios [112]. 

FcγR–pentraxin binding. Similar to FcγR-IC binding, the binding of pentraxins follows a 1:1 stoichiometry between SAP and FcγRIIa, which implies that multivalent pathogen binding is required for receptor aggregation [113].

## 4. Immunological Functions of FcγRs

The FcγR family is involved in regulating and executing antibody-mediated responses, including phagocytosis, antibody-dependent cytotoxicity, enhancing of antigen presentation, and release of cytokines and mediators of inflammation. This diversity of functional outcomes links the specificity of the adaptive immune system to the powerful effector functions elicited by innate immune cells. 

Most cells of the immune system express receptors for the constant Fc region of immunoglobulin G (IgG), which recognizes immune complexes and IgG-opsonized cells. However, it took around a decade to demonstrate the cell types that express these receptors: macrophages [7,8,14,43], monocytes [9,11], PMN [16,95], NK cells [114], B cells [12,13,15], plasma cells, basophils [19,99], and platelets [115]. T cells have been controversial; however, recently, FcγRIIb was identified in a subset of CD8+T cells (Table 1) [116]. The functional outcome resulting from the binding of immune complexes to these receptors depends on FcγR expression in the cell. The activating receptors have functions such as phagocytosis [43], antibody-dependent cell cytotoxicity [117,118], NETosis [36], enhancing of antigen presentation [119], oxidative burst [120], and release of chemoattractants. The FcγRIIb modulates cell activation, shapes the B-cell repertoire, and induces apoptosis in autoreactive plasma cells [70].

### 4.1. Phagocytosis

This process is an efficient and clean immunological host defense mechanism. Through phagocytosis, antigens immobilized with IgG antibodies are internalized and cleared. Early research on these receptors revealed their ability to induce phagocytosis through FcγRI and FcγRII in monocytes, macrophages, and neutrophils. Neutrophils constitutively express a unique combination of FcγRs: FcγRIIa and FcγRIIIb [121]. Both have a synergistic function, but FcγRIIIb alone does not generate a strong phagocytic signal [43]. However, it is known that the crosslinking of FcγRIIIb with FcγRIIa enhances phagocytosis because FcγRIIIb favors calcium influx to enhance FcγRIIa signaling [91]. The synergistic roles of both the receptors were corroborated. Recent publications have reported decreased phagocytic activity in neutrophils from FcγRIIIb-deficient donors [122]. Additionally, neutrophil FcγRIIIb crosslinking induces lipid raft-mediated activation of SHP-2, affects cytokine expression, and retards neutrophil apoptosis [123].

### 4.2. Antibody-Dependent Cellular Cytotoxicity 

ADCC allows processing of IgG-opsonized cells through FcγR. The high-affinity receptor FcγRI is only present on activated neutrophils but generally does not contribute to the ADCC of solid cancer cells, even when expressed. In contrast, FcγRIIa on neutrophils mediates the ADCC of solid cancer cells; however, FcγRIIIb restricts the antibody-dependent destruction of cancer cells. For instance, treatment with trastuzumab results in better ADCC after FcγRIIIb blockade [124]. FcγRIIIa in NK cells, macrophages, and monocytes exerts an effective ADCC, and its variants affect the monoclonal antibody activity [125].

### 4.3. NETosis

The role of FcγRs in the formation of neutrophil extracellular traps (NETs) has recently been reported. It was concluded that FcγRIIa could efficiently promote phagocytosis but could not induce NET formation on its own. In contrast, FcγRIIIb poorly promotes phagocytosis, but it can efficiently induce the formation of NETs. Note that this was concluded by testing the function of each receptor, blocking the other one. However, neutrophils express both. This information could be relevant when FcγRIIa affinity decreases and FcγRIIIb stimulation is more intense, resulting in the possibility of neutrophils with aberrant activity with a NETosis-generating phenotype [36,126]. 

NETs are a potent mechanism of defense during infections, but they are harmful in autoimmunity, NETs accelerate the inflammatory processes by releasing a wide range of active molecules, like danger-associated molecular patterns (DAMPs), histones, and active lytic enzymes (myeloperoxidase and thymidine phosphorylase) in the extracellular space, leading to further immune responses [127]. Therefore, NETs may also serve as a potential source of autoantigens (nuclear proteins in SLE) against which autoantibodies associated with SLE are directed.

## 5. FcγR Signaling Pathways 

### 5.1. Activating Signaling Pathway 

**Figure 4:**
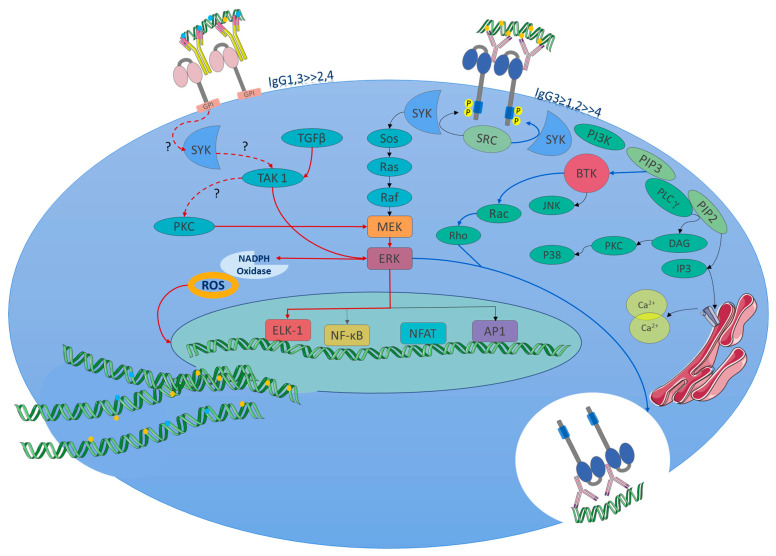
FcγRIIIb signaling pathway (NETosis pathway, red lines and arrows): Due to the lack of ITAMs, the initial steps of signaling are not yet known in detail; however, part of the signaling pathway associated with the formation of NETs has recently been described. The signaling in neutrophils of SLE patients might start from the immune complexes of autoantibodies (the figure represents autoantibody complexes with autoantigens like double-stranded DNA or nuclear proteins, which is common in SLE.) binding to the receptor. Currently, what is known about the pathway has been obtained from in vitro tests. Upon FcγRIIIb IC binding or receptor activation, the Syk and TAK 1 kinases are activated. These enzymes trigger the MEK/ERK signaling cascade. ERK signaling leads to the activation of the NADPH oxidase complex for ROS production, which is required to induce NET formation. PKC is involved in MEK/ERK pathway activation. Also, the nuclear factor Elk-1 gets phosphorylated in the nucleus by a mechanism independent of ERK. The FcγRIIIb activation promotes a pro-adhesive phenotype and enhances NETs; the contribution to phagocytosis is minimal, and phosphorylation of ERK is much more efficient in the nucleus. It favors the expression of beta 2 integrins. FcγRs signaling pathways: FcγRs activating receptors bind to immune complexes, facilitating cross-linking and intracytoplasmic activation, for which tyrosine kinases of the Src family are activated and phosphorylate tyrosine residues in ITAM on the alpha chains of the receptor. Syk, an enzyme with tyrosine kinase activity, is activated by Src. SYK phosphorylates multiple substrates, including SOS a guanine nucleotide exchange factor that activates the Ras-Raf-MEK-ERK (MAPK) pathway, which facilitates the exchange of GDP by GTP in Ras. Ras, a GTPase enzyme, activates Raf, which then phosphorylates and activates MEK, which, in turn, phosphorylates ERK. ERK activates NF-κB. FcγRIIa (Phagocytosis pathway, blue lines and arrows): Additionally, Syk activates PI3K, which produces PIP3 from the phosphorylation of PIP2 in the cell membrane, then PIP3 binds to BTK, a kinase that activates small GTPases, such as Rho and Rac. These GTPases are involved in reorganization of the actin cytoskeleton during phagocytosis. In addition, PIP3 activates PLCγ, which produces the second messengers DAG and IP3. DAG activates PKC. PKC activates the NADPH-oxidase complex to produce ROS. IP3 binds to IP3R in the endoplasmic reticulum to release Ca2^+^ into the cytoplasm. Finally, this señalización promotes a phagocytic phenotype, cytosol phosphorylation of ERK, oxidative stress, and antibody-dependent cellular cytotoxicity(depending on the cell type). Abbreviations or molecule’s function: A question mark (?) indicates an unknown mechanism of activation. Syk: spleen tyrosine kinase; TAK 1: TGF-beta activated kinase 1; MEK: mitogen-activated protein kinase; ERK: extracellular signal-regulated kinase; PKC: protein kinase C; Sos: son of sevenless (a guanine nucleotide exchange factor); Ras: a GTPase; Raf: a serine/threonine kinase; Elk-1: ETS-like gene 1, a transcription factor. BTK: Bruton’s tyrosine kinase. DAG: diacylglycerol, NF-κB: nuclear factor kappa B; PI3 K: phosphoinositide 3-kinase; PLCγ: phospholipase C gamma; IP3: inositol 1,4,5-trisphosphate. Modified from [126]. Adaptation of figure and text mechanisms [36,126,130,131,132,133].

The activating signaling pathway is partially described as the MEK/ERK pathway. It should be highlighted that FcγRIIIb signaling follows the same pathway, but with important variants, since ERK phosphorylation occurs in the nucleus when it commonly occurs in the cytosol. This differentiation allows FcγRIIIb to change the phagocytic phenotype of neutrophils to another producer of extracellular traps (in the absence of FcγRIIa activity), a distinct neutrophil phenotype recently described (Figure 4) [36]. In the context of SLE, it is important to know the triggers, receptors, and signaling pathways that lead neutrophils to form extracellular traps that contain proteins and enzymes that damage the tissue, promoting inflammation. More important these trap DNA and carry nuclear and intracellular proteins (small nuclear ribonucleoproteins) [128,129] that are recognized as autoantigens and induce the formation of autoantibodies. Following the immunological mechanism, these autoantibodies form immune complexes that bind to FcγRIIIb receptors, thereby inducing NETosis in a positive feedback loop.

### 5.2. Inhibitory Signaling Pathway 

Once the ligand (an immune complex) binds to FcγRIIb, this receptor, as an inhibitory one, contains an ITIM in the intracytoplasmic domain and recruits the inhibitory phosphatase SHIP [29], which inhibits phosphorylation of signaling molecules that have enzymatic activity, such as Btk and PLCγ, disrupting calcium flux through hydrolysis of PIP3 [134] because the necessary mediator (IP3) is not generated for binding to its receptor in the endoplasmic reticulum. 

In cells such as PMN and other innate immune cells, there is a balance between the signaling of activating Fc gamma receptors and inhibitory FcγRIIb. Therefore, the outcome of the signaling and cellular response generated depends on the binding of the immune complexes to both receptors and acting as co-signalling molecules. FcγRIIb is the sole Fcγ receptor on B cells; thus, instead of modulating FcγR activation, FcγRIIb-mediated SHIP recruitment functions primarily to modulate B-cell receptor (BCR) signaling [29].

## 6. Roles in Non-Immune Cells

### Platelets

In heparin-induced thrombocytopenia (HIT), the individual generates IgG antibodies against the chemokine platelet factor 4 (PF4), which is positively charged, while heparin is negatively charged [135]. This is a potentially dangerous immune-mediated adverse effect because it induces platelet aggregation and coagulation via FcγRIIa, leading to thrombocytopenia and thrombotic disorders [121]. Therefore, the platelet FcγRIIa receptor is a marker of increased platelet reactivity that can be reliably and repeatedly measured [136]. In addition, not only are platelets activated via the FcγRIIa receptor, but neutrophils and the immune response, through NETosis, contribute substantially to thrombosis in HIT [137].

## 7. Functions in Disease

FcγR variants and copy number variation (CNV) have been associated with autoimmune diseases; this includes systemic and organ-specific diseases. Genetic and genome-wide association studies have identified the participation of FcγR in the physiopathology of a wide variety of autoimmune diseases such as SLE [69,138], rheumatoid arthritis [139,140,141], celiac disease [140,142], and inflammatory metabolic diseases such as cardiovascular disease [143] and diabetes mellitus [140]. Additionally, the polymorphism of FcγR determines the response to treatments in cancer diseases [144].

## 8. Therapeutic Approaches 

Various therapeutic approaches related to FcγRs and their effector mechanisms have been developed. Some have been tested in animal models and others have resulted in therapeutic options already allowed and successfully used.

### 8.1. FcγRs in the Mechanism of Action of Monoclonal Antibodies (mAb)

Currently, anti-CD20 antibody immunotherapy is the most useful and representative example of a monoclonal antibody that has been extensively and exhaustively characterized. Anti-CD20 was the first mAb to effectively treat non-Hodgkin’s lymphoma and a wide spectrum of autoimmune diseases such as SLE [145], myasthenia gravis [146], neuromyelitis optica [147,148], multiple sclerosis [149,150], and pemphigus [151]. Now it is known that B cell depletion uses both FcγRI and FcγRIII-dependent pathways, and it is mediated mainly by monocytes during the anti-CD20 immunotherapy [33]. Studies in animal models and patients undergoing treatment have demonstrated that engagement of FcγRs on innate cell populations is crucial for rituximab to mediate its antitumor cytotoxic effects [152].

Also, trastuzumab (Herceptin^®^) and rituximab (Rituxan^®^) engaged both activation (FcγRIII) and inhibitory (FcγRIIb) antibody receptors on myeloid cells, thus modulating their cytotoxic potential [153].

### 8.2. Organ Transplantation

Recently, it has been reported that the inhibitory activity of FcγRIIb in a CD8+ T cell subset has a role in allograft rejection and tumor immunity. What has been observed in mouse models is the need for modulation exerted by the FcγRIIb receptor, since the intrinsic genetic deletion of FcγRIIb CD8+ T lymphocytes results in graft rejection. Additionally, when studying the influence of this receptor in a clinical trial with kidney transplant recipients, increased expression of FcγRIIb was correlated with the absence of rejection after withdrawal of immunosuppressive treatment. This is explained as follows: the Fgl2 ligand induces caspase-3/7-mediated apoptosis via FcγRIIb in CD8+ T cells, which decreases its cytotoxic action in the graft [39].

### 8.3. Recombinant Soluble Human FcγRs

Currently, the usefulness of recombinant human FcγRI, FcγRII, and FcγRIII has been studied with the aim of neutralizing the responses generated by autoantibody immune complexes in patients with autoimmunity, as the binding of these immune complexes to cell receptors promotes the activation of immune system cells, release of cytokines and inflammatory mediators, and tissue destruction. However, recombinant human Fc gamma receptors reduced IC precipitation, blocked complement-mediated lysis of autoantibody-sensitized red blood cells, and inhibited immune-complex-mediated production of IL-6, IL-13, MCP-1, and TNF-α in cultured mast cell assays. 

In addition, its efficacy against type III hypersensitivity in murine models has been tested in the Arthus skin reaction, which occurs when an antigen is injected into the skin of an individual who already has specific antibodies against that antigen. This causes the formation of antigen–antibody complexes at the injection site, leading to localized inflammation and tissue necrosis. Local or systemic administration of recombinant human FcγRIa reduces edema and neutrophil infiltration, reduces serum levels of inflammatory cytokines, and prevents paw swelling and joint damage from antibody-induced arthritis by binding to collagen [154].

### 8.4. Antibody Therapeutics: Enhancing the Inhibitory Function and Blocking the Activating Function

Early attempts to test intravenous immunoglobulin were made in the ’80s. Although the specific action mechanisms of immunoglobulin were unknown, it achieved satisfactory clinical results [155]. Engaging inhibitory FcγRIIb by the Fc region has been considered an attractive approach for improving the efficacy of antibody therapeutics. Therefore, the selective enhancement of FcγRIIb binding achieved by engineering Fc variants has provided an alternative way for improving the efficacy of antibody therapeutics [156]. The inhibition of FcγR-mediated cellular activation has been proposed as a reasonable approach to block pro-inflammatory mechanisms and immune-mediated tissue damage in autoimmune diseases. 

On the other hand, targeting FcγRIIIa (an activating receptor) with an antibody was the first promising specific therapeutic approach for an autoimmune disease [157], and following this development, several specific antibodies targeting the activating FcγRs have been developed and subjected to preclinical and clinical testing processes. Various strategies have been attempted, including the specific blocking of the main trigger receptors. However, the similarity in the sequence of the FcγR binding domains, which are an immune physiological advantage that allows the amplification of the effector functions performed by these receptors, becomes a disadvantage for the design of specific inhibitors [158]. Currently, it has been developed as blockers for FcγRI, FcγRII, and FcγRIII [158].

### 8.5. Antibody Therapeutics: Sialylation of Fc IgG to Generate Anti-Inflammatory Responses

On the other hand, more structural research has been added to improve and promote Fc-FcγR anti-inflammatory interactions. Generally, Fc-FcγR interactions generate pro-inflammatory effects of immune complexes and cytotoxic antibodies. In contrast, therapeutic intravenous gamma globulin and its Fc fragments are anti-inflammatory. It has been shown that these distinct properties of the IgG-Fc result from differential sialylation of the Fc core polysaccharide. IgG acquires anti-inflammatory properties upon Fc sialylation, which is reduced upon the induction of an antigen-specific immune response. This differential sialylation may provide a switch from innate anti-inflammatory activity in the steady state to generating adaptive pro-inflammatory effects upon antigenic challenge [159].

### 8.6. Antibody Therapeutics: Vaccines and Potentiation of Immune Response

Immunomodulatory interactions of Fc-FcγR have been leveraged as part of vaccination strategies, with the aim of eliciting broad and potent immune responses [37]. Some examples include NK cell potentiation, with the aim of preventing cancer cell resistance to NK cell-based therapy, as well as overcoming cancer cell resistance to antibody-based immunotherapy. Another strategy involves the combination of monoclonal antibodies targeting ADCC and modified NK cells to enhance the anticancer activity. Therefore, this combination enhances ADCC executed by NK cells via FcγR and allows the accumulation of effector cells in the tumor microenvironment [160].

## 9. Other Receptors for the Fc Fraction

In this review, Fc gamma receptors and their association with lupus development are addressed. However, another variety of Fc receptors is also associated with this disease, such as the Fc alpha receptor and neonatal Fc receptor, which may generate an additional perspective to expand the review at later times. A polymorphism in the coding region of FcαRI has been described, which changes codon 248 from AGC to GGC and causes a change in amino acids from G248 instead of S248 in the cytoplasmic domain of the receptor. This change affects signaling. The inflammatory G248 variant has been associated with SLE [158]. The neonatal receptor Fc (FcRn) is a protein involved in the recycling of IgG and albumin. Recent data suggest that patients with SLE have lower FcRn expression in B, NK, and T cells. In contrast, the level of FcRn was statistically higher in subpopulations of non-classical monocytes (CD14 + CD16+ monocytes) from SLE patients compared to healthy donors, providing an initial perspective to further explore its role in the pathophysiology of SLE [161].

## 10. Conclusions

Thus far, we have provided the most general information on Fc gamma receptors. More information will be added as a result of new research in the coming years, especially that related to the improvement of the response to monoclonal antibodies, which is closely related to the binding of these antibodies to activating Fc gamma receptors. Additionally, we will learn, in more depth, how FcγRs contribute to the response due to altered intestinal permeability and the consequent translocation of microbial molecules from the intestine to the blood, which increases the probability of autoimmunity, and in which scenario Fc gamma receptors mediate or modulate immune cell responses.

However, this review aimed to provide information that generates a general overview that researchers starting out in this line of research should initially know. Finally, the study of Fc gamma receptors will continue to hold more surprises. Although Fc gamma receptors are not as polymorphic as HLA, an additional advance would be to generate a database where the variants found can be added, and a systematized way of naming the new alleles, especially for alleles in which single-nucleotide polymorphisms have been considered to constitute haplotypes, as is the case for the FcγRIIIb receptor.

## Figures and Tables

**Table 1:** Human Type I FcγR Affinity and Expression in Immune Cells.

	FcγRI (CD64)	FcγRIIa (CD32a)	FcγRIIb (CD32b)	FcγRIIc (CD32c)	FcγRIIIa (CD16a)	FcγRIIIb (CD16b)
IgG_1_	6 × 10^7^	5 × 10^6^	1 × 10^5^	1 × 10^5^	2 × 10^5^	2 × 10^5^
IgG_2_	no binding	4 × 10^5^	2 × 10^4^	2 × 10^4^	7 × 10^4^	no binding
IgG_3_	6 × 10^7^	9 × 10^5^	2 × 10^5^	2 × 10^5^	1 × 10^7^	1 × 10^6^
IgG_4_	3 × 10^7^	2 × 10^5^	2 × 10^5^	2 × 10^5^	2 × 10^5^	no binding
	**Expression pattern**					
Neutrophils	●	+	+/◌	-	-	+
Eosinophils	●	+	+	-	-	●
Basophils	●	+	+	-	+	○
Mast cell	●	+	-	-	+	-
Monocytes	+	+	+	-		-
Macrophages	+	+	+	-	weak	-
NK cells	-	-	-	◌	+	-
Dendritic cells	●	+	+	-	-	-
B cells	-	-	+	-	-	-
T cells	-	-	○	-	-	-
Platelets	-	+	-	-	-	
+ constitutive expression, - no expression, ● inducible expression, ○ the expression on specific subset, ◌ depends on the allele						
**Affinity:** Neutrophils, Eosinophils, Basophils, Mast cell, Monocytes, Macrophages, NK cells, Dendritic cells, B cells, T cells, Platelets. **Expression**: Neutrophils [53,96,97,98], Eosinophils, Basophils [99], Mast cell [100], Monocytes, Macrophages [101,102], NK cells [53,103,104], Dendritic cells, B cells, T cells, Platelets. FcγRIIa exhibits a soluble form that is secreted from Langerhans cells, platelets, and megakaryocytic cell lines. It is produced by alternative splicing of transmembrane region [105], but at least in Langerhans cells has been demonstrated FcγRIIa mRNA lacking the transmembrane coding exon [106]. ○ Recent studies have identified this receptor in a subset of effector CD8+ T cells [39]. FcγRIIIb recent studies have demonstrated its expression at a low level by human basophils [99].						

## Abbreviations and Short Definitions

ADCC	antibody-dependent cell-mediated cytotoxicity
ADE	antibody-dependent enhancement
BCR	B-cell receptor
Btk	Bruton’s tyrosine kinase
CD16a	CD denomination for FcγRIIIa
CD32a	CD denomination for FcγRIIa
CD64	CD denomination for FcγRI
CNV	copy number variation
DAG	diacylglycerol
DAMPs	damage-associated molecular patterns
Elk-1	ETS-like gene 1, a transcription factor
ERK	extracellular signal regulated kinase
FcγR	Fc gamma receptor
Fgl2	fibrinogen-like 2
GPI	glycosylphosphatidylinositol
HIT	heparin-induced thrombocytopenia
IC	immune complex
IP3	inositol 1,4,5-trisphosphate
ITAM	immunoreceptor tyrosine-based activation motif
ITIM	immunoreceptor tyrosine-based inhibitory motif
kDa	kilodalton
MCP-1	monocyte chemotactic protein 1
MEK	mitogen activated protein kinase kinase
mIgG2b	mouse immunoglobulin G2
NA1	neutrophil antigen 1
NA2	neutrophil antigen 2
NET	neutrophil extracellular traps
NF-κB	nuclear factor kappa B
NSAIDs	nonsteroidal anti-inflammatory drugs
PI3K	phosphoinositide 3-kinase
PIP3	phosphatidylinositol(3,4,5)-trisphosphate
PKC	protein kinase C
PLCγ	phospholipase C gamma
PMN	polymorphonuclear leukocytes
Raf	a serine/threonine protein kinase
Ras	a GTPase
ROS	reactive oxygen species
SHIP	Src homology 2 (SH2) domain-containing inositol polyphosphate 5-phosphatase
SHP-2	SH2 domain-containing protein tyrosine phosphatase-2
SLEDAI	systemic lupus erythematosus disease activity index
SNV	single nucleotide variant
Sos	son of sevenless, a guanine nucleotide exchange factor
Syk	spleen tyrosine kinase
TAK 1	TGF-beta activated kinase 1
VEGF-A	vascular endothelial growth factor
Aminoacid Abbreviations	
E	glutamic acid
F	phenylalanine
H	histidine
I	isoleucine
L	leucine
Phe	phenylalanine
R	arginine
Se	serine
T	threonine
